# Multiple Intracranial Tuberculomas with an Intra-medullary Spinal Cord Tuberculoma in a Pediatric Patient

**DOI:** 10.7759/cureus.7248

**Published:** 2020-03-12

**Authors:** Sreenath Meegada, Richmond Gyamfi, Vijayadershan Muppidi, Vasuki Dandu, Tejo Challa

**Affiliations:** 1 Internal Medicine, The University of Texas Health Science Center/Christus Good Shepherd Medical Center, Longview, USA; 2 Internal Medicine, Prevea Health, De Pere, USA; 3 Internal Medicine, Indiana University Health, Indianapolis, USA; 4 Neurology, Baptist Health Medical Center, Little Rock, USA

**Keywords:** cerebral tuberculomas, disseminated tuberculosis, intracranial tuberculoma, intra-medullary spinal cord tuberculoma, central nervous system tuberculosis

## Abstract

Central nervous system (CNS) tuberculosis (TB), caused by Mycobacterium tuberculosis (MT), is a severe form of TB, which presents as meningitis, cerebritis, abscesses, spinal tuberculous arachnoiditis, and rarely tuberculomas. CNS TB is prevalent in the underdeveloped or developing world and is common in malnourished, alcoholics, children, young adults, immunocompromised, and cancer patients. Intracranial tuberculomas (ICT) can present with symptoms and signs of focal neurological deficits with or without systemic manifestations. ICT is the least common presentation of CNS TB. Medical management with anti-TB drugs and steroids is the mainstay of treatment, while surgical intervention is usually reserved for refractory cases. Here, we present the case of a 10-year-old Indian American girl with headaches, diplopia, fever, and neck pain diagnosed with ICT and intramedullary spinal cord tuberculoma.

## Introduction

Central nervous system (CNS) tuberculosis (TB) is a rare form of TB due to the hematogenous spread of Mycobacterium tuberculosis (MT) from the primary site [1]. It continues to be a prevalent endemic problem in underdeveloped and developing nations [1]. It accounts for 1% of all TB cases, and 5% to 10% of extrapulmonary TB cases. Globally, an estimated 10 million people fell ill with TB per the World Health Organization (WHO) 2019 report [2]. CNS TB carries high morbidity and mortality, which warrants early diagnosis and treatment. A definitive diagnosis of CNS TB is made by the detection of TB bacilli in cerebrospinal fluid or tissue cultures. Risk factors for CNS TB include children, malnutrition, alcoholism, malignancies, and immunosuppressed states [3].

## Case presentation

A 10-year-old Indian American girl born in the United States presented with a two-week history of intermittent headaches, fever, neck pain, and transient double vision. She described her headache as global, intermittent, throbbing in nature, non-radiating, with no relieving factors. She had a fever of 103 °F on the day prior to admission. Neck pain was constant, with a decreased range of motion at the neck. She also had a transient double vision, which lasted for a few hours. She lost 7 pounds in two months. She did not have any sick contacts and was otherwise healthy. Upon questioning, she revealed travel history to India six months ago to visit her grandparents for the first time. She denied sick contacts in India during her stay. 

**Figure 1:**
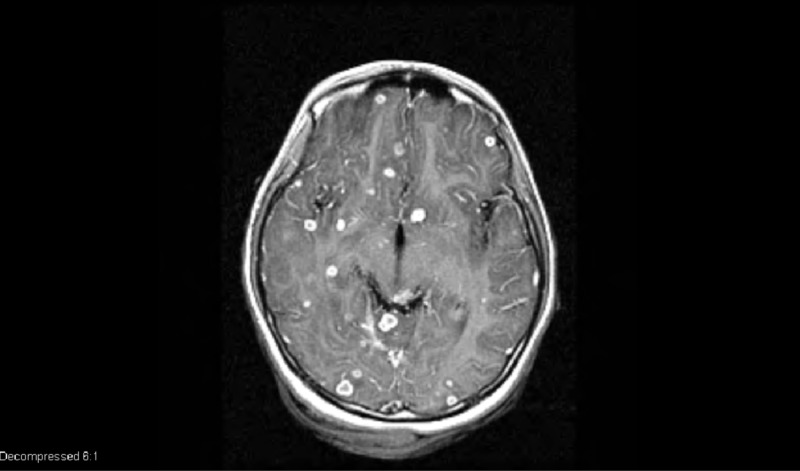
Magnetic resonance imaging (MRI) of the brain with gadolinium contrast showing multiple ring enhancing lesions

In the emergency room (ER), the patient was feeling tired, fatigued. Vital signs were normal with no fever. Heart rate, respiratory rate, and blood pressure were normal. On physical exam, the patient was a weak, tired-looking girl. The lungs were clear; heart was normal with sounds, no murmurs; abdomen was soft and benign; eye exam was normal with extra-ocular movements intact; oropharynx was moist, no erythema, normal-looking tonsils. The neurological exam demonstrated normal reflexes, tone, no cranial nerve palsies; the cerebellar system was intact. Lab workup was normal; chest X-ray was normal with no infiltrates; computed tomography (CT) scan of the head showed some multiple suspicious lesions, after which magnetic resonance imaging (MRI) scan of the brain with contrast was done which was positive for multiple ring-enhancing lesions throughout the brain (Figure 1).

**Figure 2:**
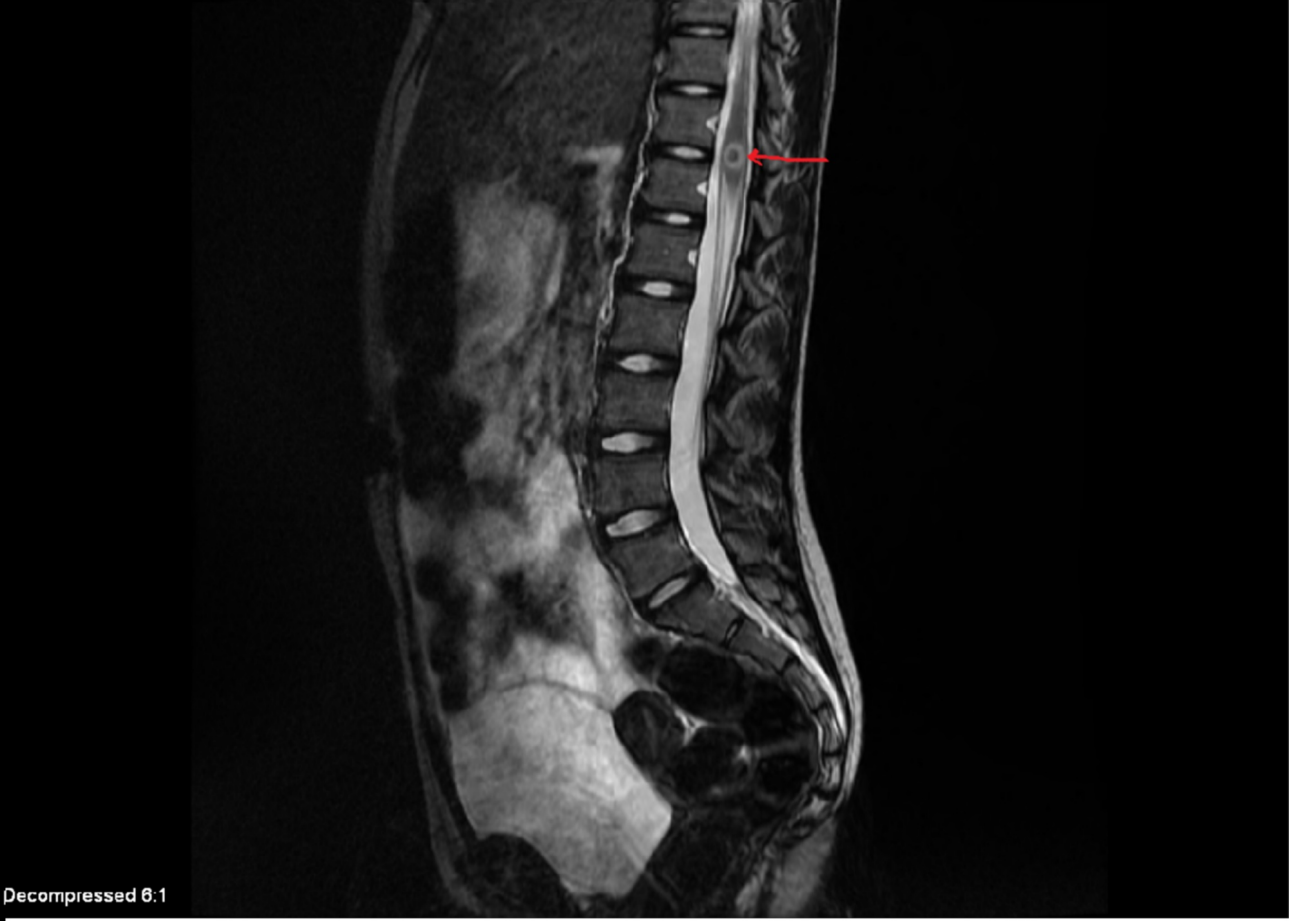
Magnetic resonance imaging (MRI) of the spine with gadolinium contrast showing 9-mm ring enhancing lesion (red arrow)

MRI spine revealed a 9-mm enhancing lesion too (Figure 2).

**Figure 3:**
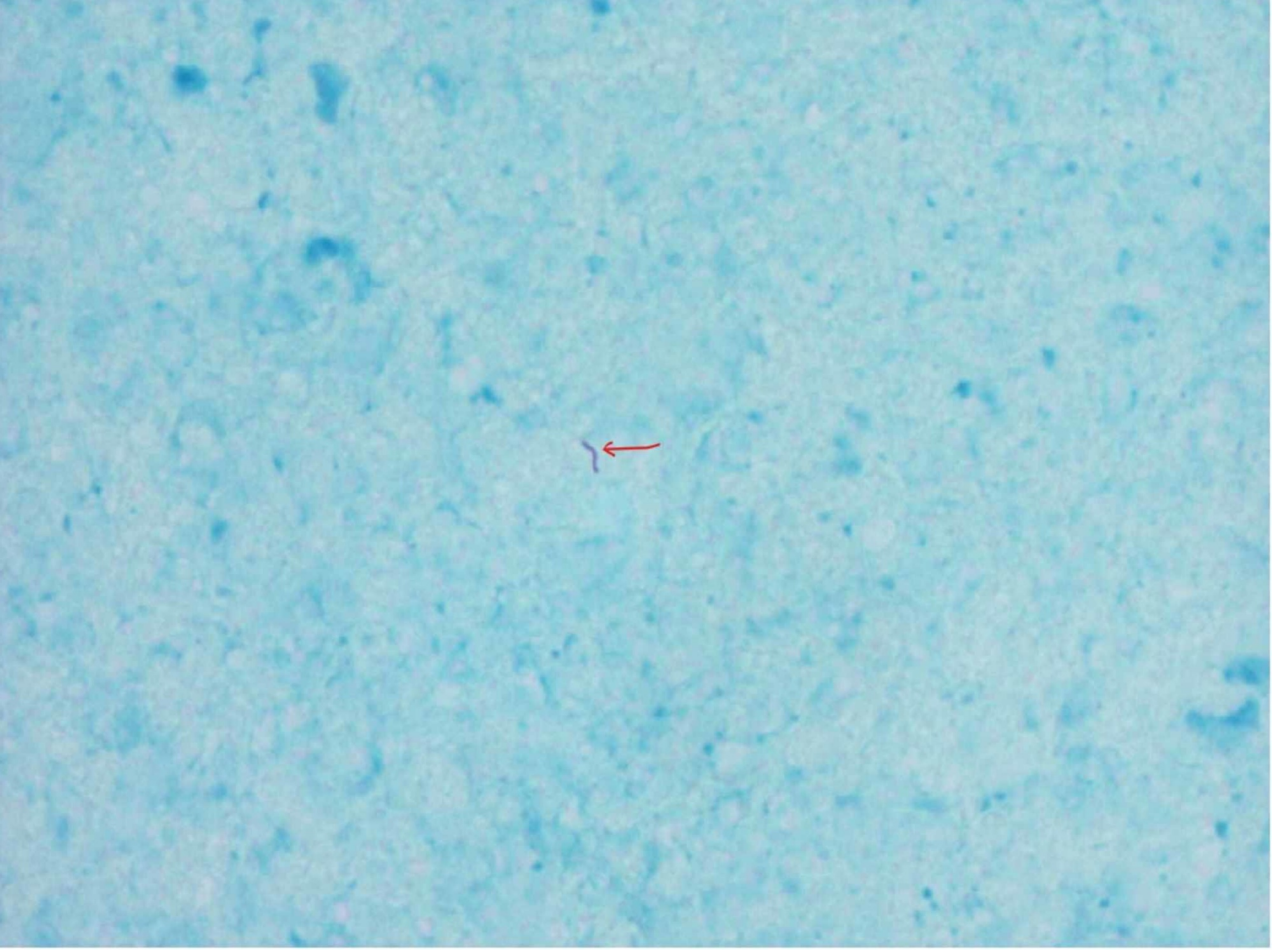
Biopsy tissue revealed acid-fast stain positive for bacilli (red arrow)

Considering her travel history to India, CNS tuberculoma was high on the differential. She had stereotactic brain biopsy, which was acid-fast stain positive (Figure 3).

**Figure 4:**
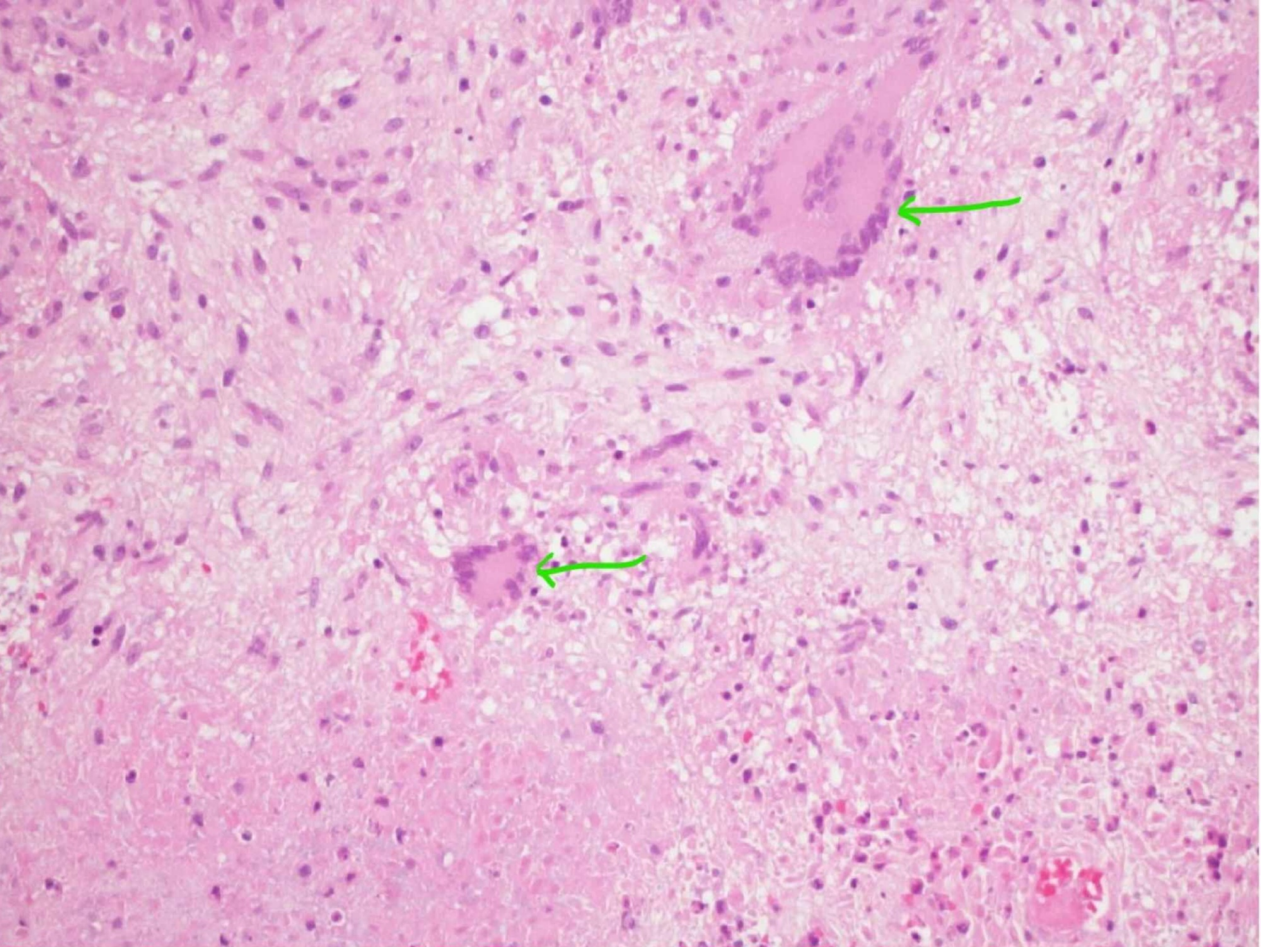
Caseating granulomas seen on hematoxylin and eosin stain (green arrows)

Histopathology showed caseating granulomas (Figure 4).

**Figure 5:**
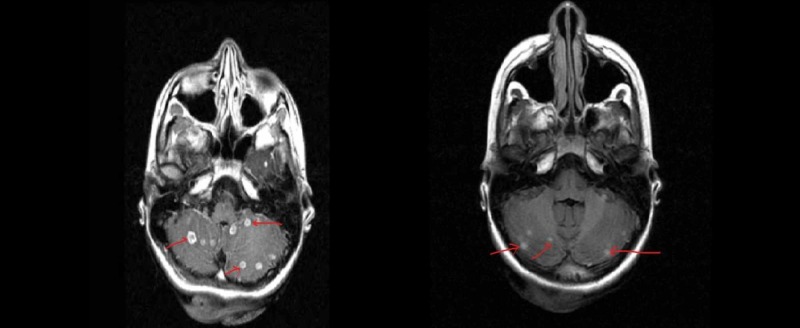
Pre- (left) and post-treatment (right) magnetic resonance imaging (MRI) scans of the brain showing decreasing lesions (red arrows)

**Figure 6:**
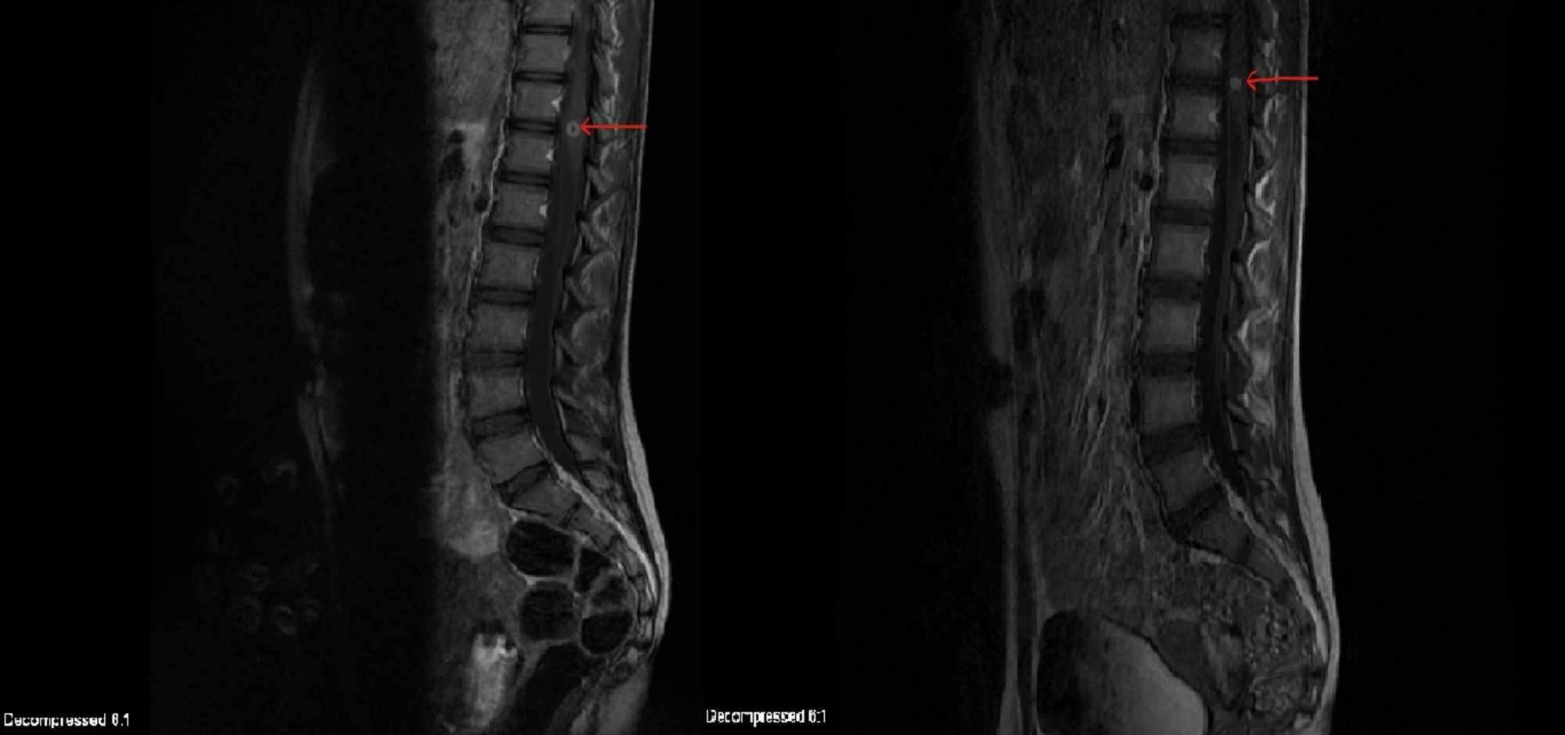
Pre- (left) and post-treatment (right) magnetic resonance imaging (MRI) scans of the spine showing decreasing size of spine lesion (red arrows)

The culture grew MT, which was sensitive to conventional anti-TB drugs. She was initiated on four drugs (isoniazid, rifampin, pyrazinamide, ethambutol) and steroid taper with significant improvement in her lesions after five months of treatment, which was evidenced in repeat MRIs (Figures 5-6).

## Discussion

CNS TB presents clinically as TB meningitis, spinal TB arachnoiditis, abscesses, and rarely as tuberculomas. Pediatric patients infected with TB rarely (2%) present with tuberculomas. Because of its rarity, non-specific presentation, and radiological findings, early diagnosis is still a clinical challenge [4]. TB infection occurs through inhalation of a droplet containing bacilli, which leads to the deposition in the lung alveoli and activation of T-helper cells. This T-helper cell immune-mediated response leads to the formation of granuloma and primary pulmonary TB. Prior to the containment of the infection, some bacilli are filtered into the lymphatic system, which leads to hematogenous dissemination to the distant parts of the body, which are highly oxygenated like the brain. A multitude of factors like host immunity, the virulence of TB strain, and oxygenation plays a vital role in clinical features of TB [5].

ICTs can present with headache, fever, seizures, hemiplegia, papilledema, or occasionally with signs of raised intracranial pressure and non-specific features like fatigue, weight loss, lack of appetite [6,7]. Our patient presented with fever, headache, transient diplopia, and neck pain. Clinical manifestations usually correlate to the location of tuberculoma in the brain. Meningeal signs and symptoms of systemic illness are not commonly observed. Diagnostic evaluation of ICT starts with proper history taking including travel history, physical exam with thorough neurological examination, sputum acid-fast bacilli (AFB) stain, culture and sensitivity, chest X-ray, TB skin test, QuantiFERON gold test (Cellestis Limited, Carnegie, Victoria, Australia), CT scan of the brain/chest, and MRI of the brain [8,9]. Definitive Diagnosis of ICT is usually made by demonstration of TB bacilli in the biopsied tissue [10].

Treatment is started based on strong clinical suspicion and should not be delayed because of the time involved (4-8 weeks) in obtaining TB culture and sensitivities [11]. The treatment regimens are outlined and published in the United States Centers for Disease Control and Prevention (CDC) [12]. CNS TB is usually treated with an initial intensive phase of four drugs (isoniazid, rifampin, pyrazinamide, and ethambutol) for two months, followed by a prolonged continuation phase of two drugs (isoniazid and rifampin) for additional 7-10 months. Treatment must be tailored to the culture and sensitivity of the TB bacilli and drug resistance. According to the American Academy of Pediatrics (AAP), ethionamide or streptomycin can be a substitute for ethambutol in children where ethambutol induced optic neuritis cannot be monitored [13]. Adjunctive steroid therapy is recommended in patients with TB meningitis (suspected or confirmed), basilar enhancement on radiographic imaging, presence of intracranial tuberculomas (like our patient), cerebrospinal fluid protein greater than 500mg/dL, and worsening of clinical signs after being on anti-TB therapy [14-16]. Surgery is only reserved for patients not responding to medical therapy, obstructive hydrocephalus, midline shift, and compression of the brain stem or spinal cord [17,18]. Unlike CNS mass lesions, surgical resection of tuberculomas is associated with severe fatal meningitis. Hence medical management is recommended as first-line management.

## Conclusions

CNS tuberculoma should be considered in TB patients presenting with neurological deficits or raised signs of intracranial pressure and with no symptoms of systemic illness or meningeal signs. Early diagnosis and treatment are crucial in CNS TB, considering its high morbidity and mortality. CNS tuberculoma should be considered in the differential diagnosis of ring-enhancing lesions on brain imaging in patients with risk factors, as mentioned. Treatment is with conventional anti-TB regimen along with steroids. Surgical resection is rarely needed.
